# Preference bias of head orientation in choosing between two non-durables

**DOI:** 10.3389/fpsyg.2015.00849

**Published:** 2015-06-23

**Authors:** Hiroyuki Funaya, Tomohiro Shibata

**Affiliations:** Graduate School of Life Science and Systems Engineering, Kyushu Institute of TechnologyKitakyushu City, Fukuoka, Japan

**Keywords:** head orientation, choice prediction, real-shop marketing, forced choice between two alternatives, preference bias

## Abstract

The goal of this study is to investigate how customers' gaze, head and body orientations reflect their choices. Although the relationship between human choice and gaze behavior has been well-studied, other behaviors such as head and body are unknown. We conducted a two-alternatives-forced-choice task to examine (1) whether *preference bias*, i.e., a positional bias in gaze, head and body toward the item that was later chosen, exists in choice, (2) when *preference bias* is observed and when prediction of the resulting choice becomes possible (3) whether human choice is affected when the body orientations are manipulated. We used real non-durable products (cheap snacks and clothing) on a shopping shelf. The results showed that there was a significant *preference bias* in head orientation at the beginning 1 s when the subjects stood straight toward the shelf, and that the head orientation was more biased toward the selected item than the gaze and the center of pressure at the ending 1 s. Manipulating body orientation did not affect the result of choice. The *preference bias* detected by observing the head orientation would be useful in marketing science for predicting customers' choice.

## 1. Introduction

Human behaviors in choice have been extensively studied in various fields. Payne (1976) proposed a two-stage choice process that was later extended by Russo and Leclerc (1994) who divided human choice process into three stages by observing gaze behaviors. Gaze has been specially investigated in a two-alternative-forced-choice (TAFC) paradigm in neuroscience (Carpenter and Williams, 1995), psychology (Krajbich et al., 2010; Orquin and Mueller, 2013) and marketing science (Ratcliff and Smith, 2004; Sorensen, 2009). There is a gaze *cascade effect* in which the position of gaze is biased toward the selected item before the decision is made in TAFC tasks (Shimojo et al., 2003; Bird et al., 2012). In the *cascade effect*, the human gaze gradually shifts toward what is eventually chosen before the decision moment, which would be useful in marketing science for predicting customers' choice. Gaze *cascade effect*, however, refers to the gaze behaviors immediately before the decision, and the relationship between preference and other human behaviors such as face or body orientation were out of scope.

In marketing science, Sorensen (2009) divided common shopping behaviors into three stages: reach, stop and close and studied the customers' moving paths in a supermarket. Other studies also explored the walking path to analyze its relations to the resulting purchase (Yada, 2011; Rao and Chandran, 2013). Our interest in this study is how customers behave after stopping at the shelf. Therefore, we asked subjects to stand on a shopping shelf and not to move from the initial position while we allowed them other movements of gaze, head, and torso, etc.

The purposes of this study were to investigate (1) whether *preference bias*, i.e., a positional bias in gaze, head and body toward the item that is later chosen, exists in a TAFC task involving non-durable goods with different values, (2) when *preference bias* is observed and when prediction of the resulting choice becomes possible. We used real products closely placed side by side on a shopping shelf to evaluate human behaviors in a more realistic setting. To further investigate (3) whether human choice is affected by the body orientation relative to the items, we additionally manipulated subjects' body angles during choices and analyzed its correlation to the resulting choices. In manipulating body orientation, the bias stemming from body orientation was subtracted from the total bias to extract *preference bias*.

## 2. Materials and methods

### 2.1. Subjects

We employed healthy subjects (*n* = 16, 13 males and 3 females, all Japanese, age 22–28). We asked subjects who usually wear glasses to remove them so that they could wear an eye tracker. In such cases, we confirmed that they had no problem seeing the items on the shelf without their glasses. They all agreed to the ethical engagements defined by the local committee of the Kyushu Institute of Science and Technology.

### 2.2. Experimental setup

**Figure 1:**
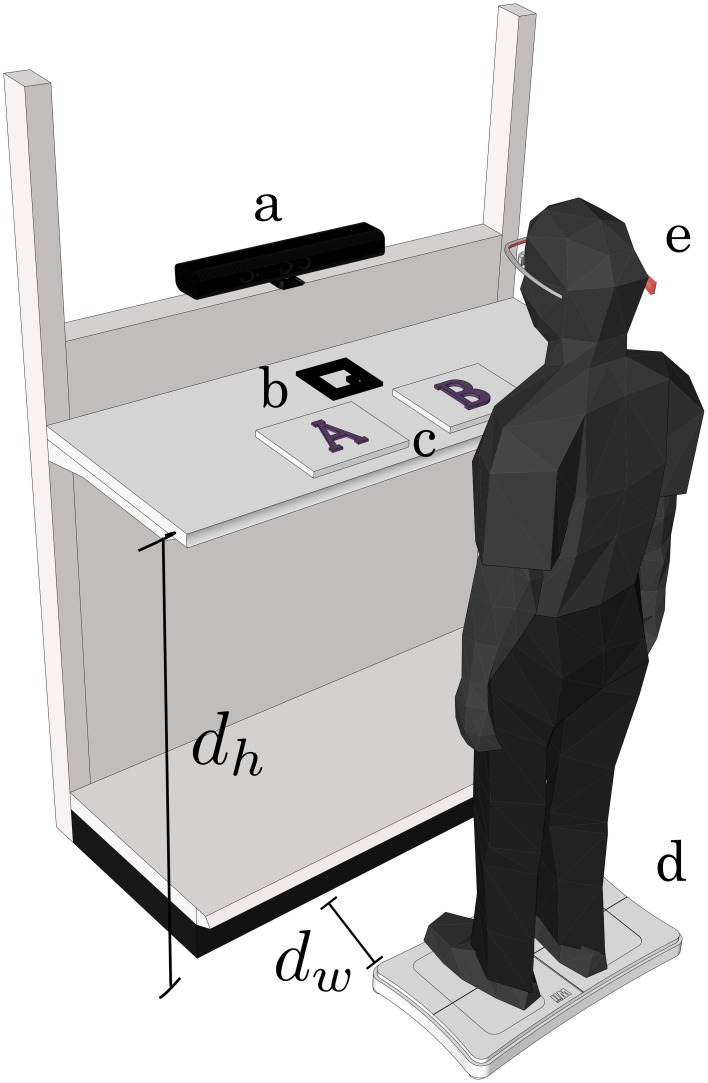
**Experimental setup of shelf: a, webcam; b, AR marker for locating of the center of the two alternatives; c, items horizontally aligned; d, Wii Balance Board (WBB); and e, eye tracker (EMR-9)**. Shelf top height *d_h_* = 98 (cm) and distance between WBB and shelf *d_w_* = 35 (cm). The subject wearing the eye tracker stands on a WBB after the operator gives a cue. The shelf was a commercial shelf used in real stores.

Figure 1 shows an overview of the experimental setup. The presented items were cheap non-durables from two categories: snacks (worth 1–2 U.S. dollars) and clothing (worth 5–20 U.S. dollars). In total 18 pairs of snacks and cloths were prepared while 12 pairs were randomly chosen for every experiment. The remaining 6 pairs were used for instruction and for operational mistakes such as invalid recordings that were found during the experiments. Each pair consisted of two items similar in size and appearance, e.g., chocolates of different flavors, pairs of socks, T-shirts of the same size, etc. The size of the items varied from small chocolates (5 × 12 [cm]) to large sweaters (30 × 40 [cm]). The distance between the left edge of the right item and the right edge of the left item was kept under 20 [cm].

We are interested in the body movements in addition to eyes and faces as the indicators of human preference. We measured the angle of upper body (torso) by an external depth sensor Microsoft's Kinect™ and its SDK during the choice behavior. It however was too noisy to track the movement of torso because of the precision of the SDK's tracking algorithm (Obdrzalek et al., 2012). Instead, a Wii Balance Board (WBB; Nintendo, Kyoto, Japan) was employed to evaluate the preference bias in the subject's center of pressure (COP). Note that WBB has a sufficient accuracy for assessing standing balance (Clark et al., 2010). The WBB was set in front of the shelf and a chair was provided in front of the WBB for subjects to rest on between experiments. The subject wore a portable eye-tracking system, the EMR-9 (NAC, Tokyo, Japan) while standing on the WBB. The subjects were allowed to move their body freely instead of their feet on the WBB. A flat marker was placed on the shelf for later image processing of the EMR-9's head-mounted camera. The height of the shelf *d_h_* and the distance between the shelf and WBB *d_w_* were kept constant throughout the experiments. A webcam was installed to synchronize the data from the WBB and EMR-9. The whole setup was covered by thick cloth to avoid presenting unwanted visual stimuli to the subjects. Two normal lamps above the shelf were installed to control the light level.

### 2.3. Experimental protocol

Each subject had 12 trials each of which had a different pair of items. They were asked to select one of the pairs with no time restriction. The response time was defined as the duration from when the subject started observing the items to when the subject made a decision. To know the decision timing, we asked the subjects to point to the item they preferred with their finger. Unlike previous studies, we did not employ a physical button, in order to eliminate unnatural choice behaviors that would not occur in a real-shop situation.

Before each trial began, the whole setup shown in Figure 1 was kept dark with the lights turned off. The subject sat on the chair in front of the WBB, and was asked to stand up on the WBB's footprint mark and look straight ahead. The subjects were asked to pay no attention to the shelf top before each trial's start. A trial began when the operator turned the light on and started recording all the signals (WBB, webcam and EMR-9). At the same time, the subject started looking at the pair of items. The operator monitored the subject outside the booth with the webcam until the subject pointed to either of the items. Finally, the operator told the subject to sit back down on the chair, turned the lights off and changed the items for the next trial.

Each subject performed 12 trials executed in a pseudo-random order as shown in Table 1: three snack trials, three apparel trials, then the same numbers of trials in the same order. The trial categories were fixed, but the pairs of products were randomized with no duplication. Also, we did not fix the sides of the alternatives to avoid bias stemming from left-right alignment.

**Table 1:** **Pseudo-random item categories and WBB angles in the 12 trials**.

**Trial Number**	**1**	**2**	**3**	**4**	**5**	**6**	**7**	**8**	**9**	**10**	**11**	**12**
Category	S	S	S	C	C	C	S	S	S	C	C	C
Angle	s	l	r	s	l	r	s	l	r	s	l	r

*S, snack; C, clothing; s, straight; l, left; r, right*.

**Figure 2:**
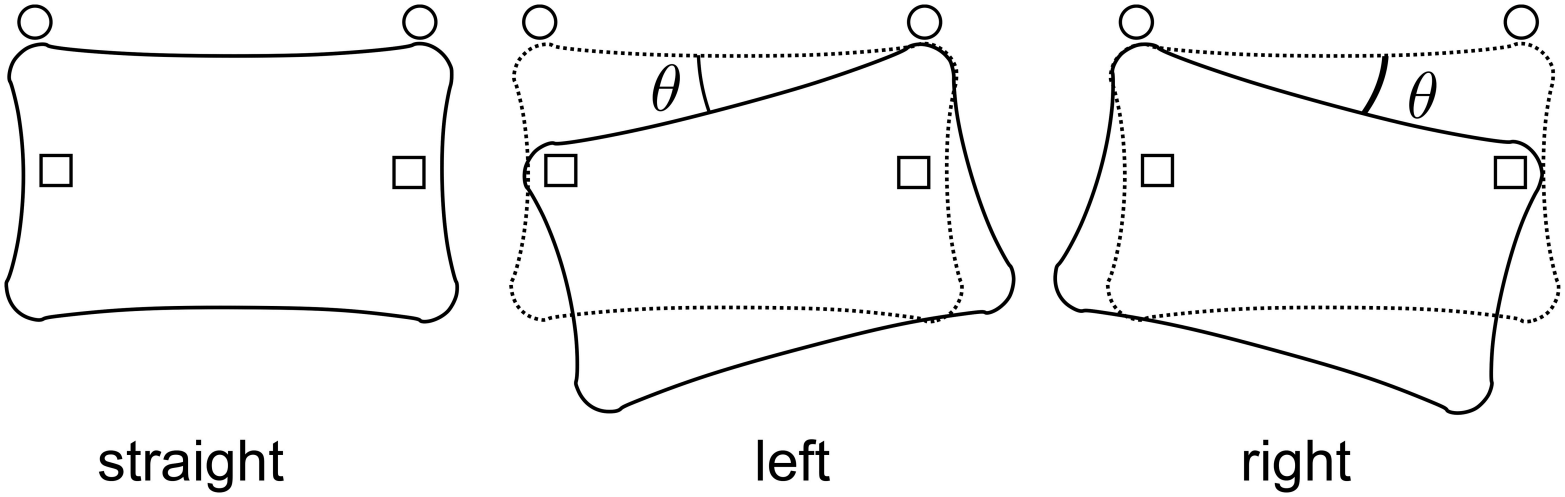
**Body orientation manipulation: the WBB was set at three different angles**. Circles and squares in the figure represent the markers placed on the floor. Because the board was manually set by the operator, there are small variations, but θ ≃ arctan(9/48) ≃ 10.8 [degrees], which was calculated from the relative marker locations.

We manipulated the angle of the WBB to study the effect of body-orientation on final choice (Figure 2). We tested three angles, *left*, *right*, and *straight*, relative to the longer edge of the shelf, and we switched the angle after every trial as shown in Table 1.

### 2.4. Data collection and preprocessing

First, the data from the webcam and EMR-9 were synchronized by observing when the lights were turned on. The data from the webcam and the WBB were recorded on the same PC and synchronized in advance. The start time of each trial was marked in both the webcam's and the EMR-9's views by observing at least one item present in the view of the head-mounted camera.

We were interested in if and when each sequence (head orientation, gaze, and COP) is biased toward the selected item, but it was difficult to handle the sequences in an unified way because the current experiment was designed as a time-free choice. Hence, we synchronized the data at the starting and at the ending timings of each trial. The starting time was when at least one item first appeared in the view of the eye-tracker's camera and the ending time of each trial was marked when the subject's arm starts moving his or her arm to point the selected item. The data were averaged over first and last 1 s and tested if each signal was biased toward the selected items. This partly follows previous time-free studies such as Shimojo et al. (2003) in which they also synchronized the data at the end of each trial and discussed the behaviors of last 1 s. To eliminate any overlap between the beginning and ending 1 s, short trials whose response time were under 2 s were omitted from the analysis.

The types of collected signals were head orientation, gaze, and COP, denoted by *s_h_*(*t*), *s_g_*(*t*), and *s_p_*(*t*), respectively, where *t* is the elapsed time from when at least one item first appeared in the view of the head-mounted camera. Because the items were arranged horizontally, we tracked only the horizontal movement of those signals. *s_h_*(*t*) and *s_g_*(*t*) were defined in the view of the head-mounted camera (“head view” hereafter) and so the units were pixels, while *s_p_*(*t*) was defined within dimensions of the WBB surface and its unit was millimeters (**Figure 4**).

**Figure 3:**
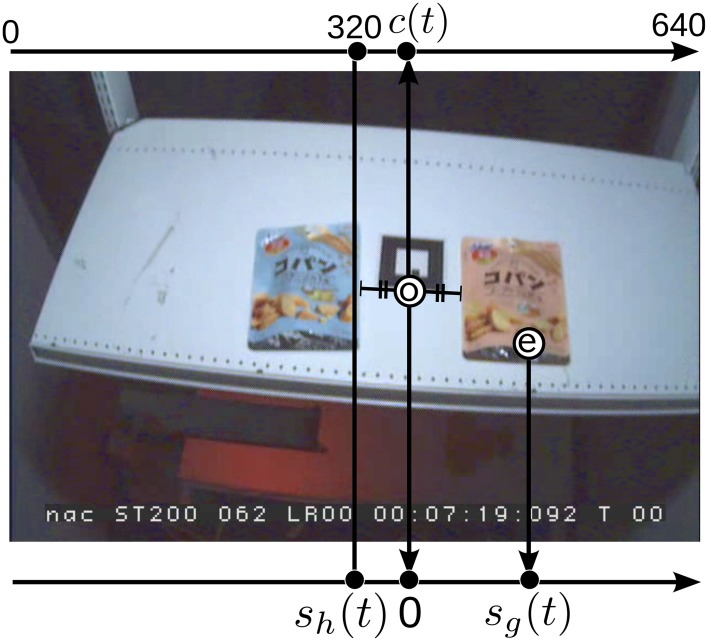
**Definition of head center *s_h_*(*t*) and gaze *s_g_*(*t*) in the head-mounted camera's view: The item center *o* is semi-manually tracked and *c*(*t*) is calculated on the axis that starts from the left edge (shown on the top of the image)**. Then, *s_h_*(*t*) and *s_g_*(*t*) are defined by the coordinate axis shown at the bottom of the image where *c*(*t*) was set as the origin. The circled *e* is the gaze point reported by the eye tracker. Note that the sign of *s_h_*(*t*) is positive when the head is oriented to right and negative for the left orientation.

Figure 3 illustrates how we calculated *s_h_*(*t*) and *s_g_*(*t*). *s_h_*(*t*) was defined as the point at which the imaginary normal vector of the subject's face intersected the shelf surface. To calculate *s_h_*(*t*) and *s_g_*(*t*), we needed to track the center of the items in the head view. To do so, we first manually tracked the center of the items (also in Figure 3) every 2 s throughout a trial by mouse clicking. Then, marked points were interpolated by applying a global Lucas-Kanade optical flow (Lucas and Kanade, 1981) and applying a tracking method called *good features to track* (Tomasi and Shi, 1994). These algorithms allowed us to find identical points in the two images and to calculate whole view shifts in adjacent frames.

**Figure 4:**
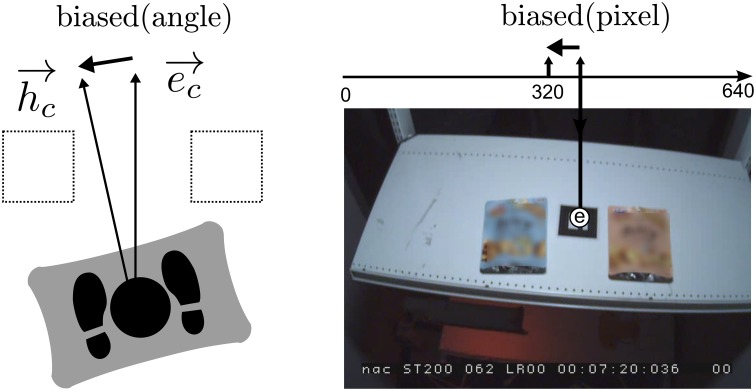
***Body-orientation*** **bias in real world coordinates (left) and head-mounted view (right): The body-orientation bias can be observed in the coordinates of the head-mounted view**.

The horizontal value of COP, *s_p_*(*t*) [mm], was calculated based on the values of four weight sensors sent from the WBB as in Bartlett et al. (2014). *s_p_*(*t*) = 0 indicates that the same pressure was put on both feet.

### 2.5. Body-angle manipulation

To test the effect of the angle manipulation, we first counted the number of trials in which the side of the selected item and the manipulated angle were matched. Then, we calculated the rate of such trials to the number of the manipulated trials (angle conditions were *left* or *right*) for each subject. Finally, we tested whether the rates were statistically biased from the chance level (see Results Section).

### 2.6. Preference bias

In this study, we investigated if each signal toward the selected item

(1) $$\pi (t)=\{\begin{array}{ll} \;s(t) & \text{if left item was selected}\\ -s(t) & \text{otherwise}, \end{array}$$

was biased. We defined π_*h*_(*t*), π_*g*_(*t*), and π_*p*_(*t*) using *s_h_*(*t*), *s_g_*(*t*), and *s_p_*(*t*) for gaze and COP, respectively. To obtain robust properties of *preference bias*, the data of each sequence were averaged over the beginning 1 s and resulting values were averaged over all the trials for each subject. Namely, we have the same number of samples as that of subjects in the statistical tests described below. We applied the same averaging process for the data of the ending 1 s.

After the independent tests, the differences between three types of signals (head orientation, gaze, and COP) were assessed. To do so, normalization was needed because human gaze directly points the item in most cases while the head does not necessarily direct it. As a consequence, the absolute value of gaze tends to be larger than that of head orientation. Also, the COP was measured in the surface of the WBB while other two signals were measured in the view of the eye-tracker's camera. Therefore, to compare the differences between those signals, each sequence was first divided by its maximum absolute value. This is because the observed signals varied among subjects and trials due to personal tendencies and sizes of the stimuli. The normalized signals were then compared using the Steel-Dwass method.

### 2.7. Body orientation bias

*s_h_*(*t*) can be affected by body orientation. Funatsu et al. (2013) studied how much head orientation changes when they manipulated body orientation relative to a static object. They found that there is a linear relationship between body orientation and head orientation. In the current experiment, the signals may be biased by manipulating body orientation. Because this *body-orientation bias* we here define is assumed to be irrespective to *preference bias*, it was subtracted from the observed signal as the following procedure.

We redefined each signal s_*_(*t*) as *s*^a^_*_(*t*) where * means one of *h*, *g* or *p* and *a* ∈ {*left*, *right*, *straight*} specifies the trial condition of the WBB angle. For example, when the WBB was in the angled-right position, *s_h_*^*right*^(*t*) was right-biased from the center position of the items, while the centers match when the WBB is not angled. We define *Body-orientation bias* as

(2) $$\beta_{*}^{a}=\frac{1}{tn}\sum_{t}\sum_{n}s_{*}^{a}(t,n)\;(a\in left,straight,right),$$

where *n* is the index of each trial. In other words, *body-orientation bias* is the constant biases in gaze, head orientation, and COP averaged over all trials of the same angle condition of a subjects' body orientation. We here assumed that *body-orientation bias* was a constant during a trial because the subject's feet were fixed in this experiment, and assumed that all subjects have the same β^*a*^ for each angle condition *a*. We tested for the existence of *body-orientation bias* before calculating *preference bias* and subtracted those biases when they existed.

The total bias was defined as the simple addition of *preference bias* and *body-orientation bias*. Hence, we defined the signal for body orientation as

(3) $$\pi_{*}^{a}(t)=\{\begin{array}{ll} \;s_{*}^{a}(t)-\beta_{*}^{a} & \text{if the left item was selected}\\ -\left(s_{*}^{a}(t)-\beta_{*}^{a}\right) & \text{otherwise}, \end{array}$$

similar to Equation (1). Similar to the analysis of *preference bias*, we averaged all the trials for each subject and obtained one sample per subject. To test whether *body-orientation* bias exists, we applied one-way analysis of variance for the samples of three angle conditions.

In the analysis of *preference bias*, we considered two cases without (*case 1*) and with (*case 2*) subtracting *body-orientation bias*. That is, in *case 1*, only the trials with *a* = *straight* were used while all the trials were used after subtracting *body-orientation bias* in *case 2*.

## 3. Results

For the analysis without *body-orientation bias*, 54 out of 192 trials (*a* = *straight*) were used as *case 1* and 172 out of 192 trials were used as as *case 2* after considering *body-orientation* bias. Short trials that finished within 2 s were omitted. Other trials with operational and system errors were also omitted. Because each sample was obtained by averaging all the trials per subject, the total number of samples became *n* = 15 in *case 1* and *n* = 16 in *case 2*. One sample was removed in *case 1* because all four trials of one subject with angle condition *a* = *straight* finished within 2 s.

First, we calculated the ratio of the side of selected items per subject (number of selected items/total number of valid trials) and compared those among the conditions of body angles, but there was no significant evidence that manipulation of body angle affected the side of the selected item. (*p* = 0.8, one-sample Student's *t*-test with parameter μ = 0.5, 15° of freedom).

Next, we collected the body-orientation biases β_*_ averaged over all trials per subject. There were no body-orientation biases in *s_g_*(*t*) and *s_p_*(*t*), but *s_h_*(*t*) was biased significantly (*p* = 0.6, *p* = 0.6, and *p* < 10^−6^, respectively, Kruskal-Wallis test after Shapiro-wilk normality test) to the angled direction. In the case of *s_p_*(*t*), there was no significant bias in the angle condition *a* = *straight* (*p* = 0.2, one-sample Student's *t*-test with mean parameter μ = 0) while there were biases in the case of *a* = *left* and *a* = *right* (*p* = 0.01 and *p* = 0.0002, respectively, same tests as above). Therefore, we set β^*left*^_*h*_ = −38.4 and β^*right*^_*h*_ = 40.3, while the other biases were treated as zero in Equation (3). The degrees of freedom of the tests above were all 15.

**Figure 5:**
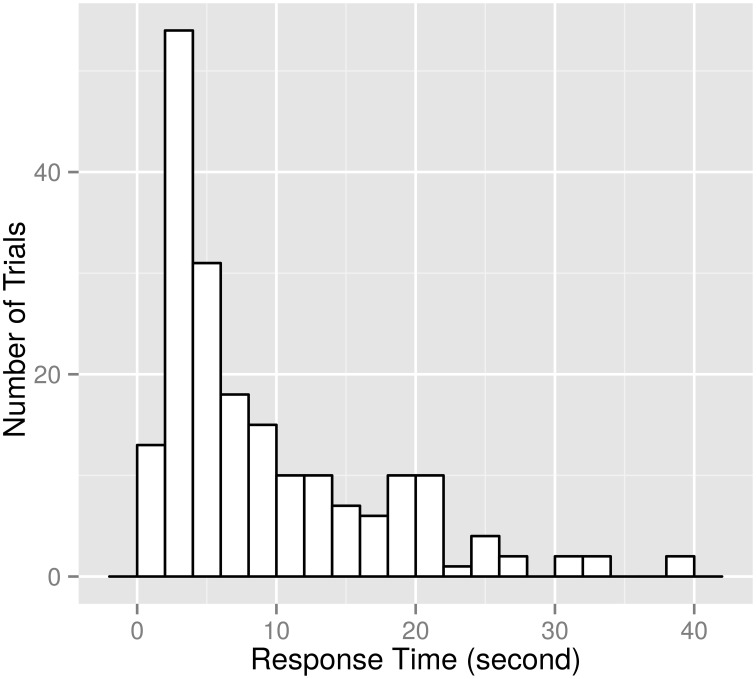
**Histogram of response times of 172 trials (*****case 2*****)**. The horizontal axis shows response time in seconds. The vertical axis shows the number of trials. Each bin has a constant width of 2 s. Seven trials in *case 2* and four trials in *case 1* had response times less than 2 s, and thus we omitted those trials from the analysis.

Figure 5 shows the histogram of the response times for *case 2*. As described in the Materials and Methods Section, the starting time was when at least one item first appeared in the view of the eye-tracker's camera and the ending time was when the subjects started moving their arm to point what they like. We collected the response times averaged over corresponding trials (typically 4 in *case 1* and 12 in *case 2*) per subject. There were no significant difference in response times for the item categories (*p* = 0.8 in *case 1* and *p* = 0.8 in *case 2*, Student's *t*-test). Also, which side the selected item was on (left or right) did not affect response times (*p* = 0.8 in *case 1* and *p* = 0.7 in *case 2*, Student's *t*-test). The degrees of freedom of the tests above were 14 in *case 1* and 15 in *case 2*.

**Figure 6:**
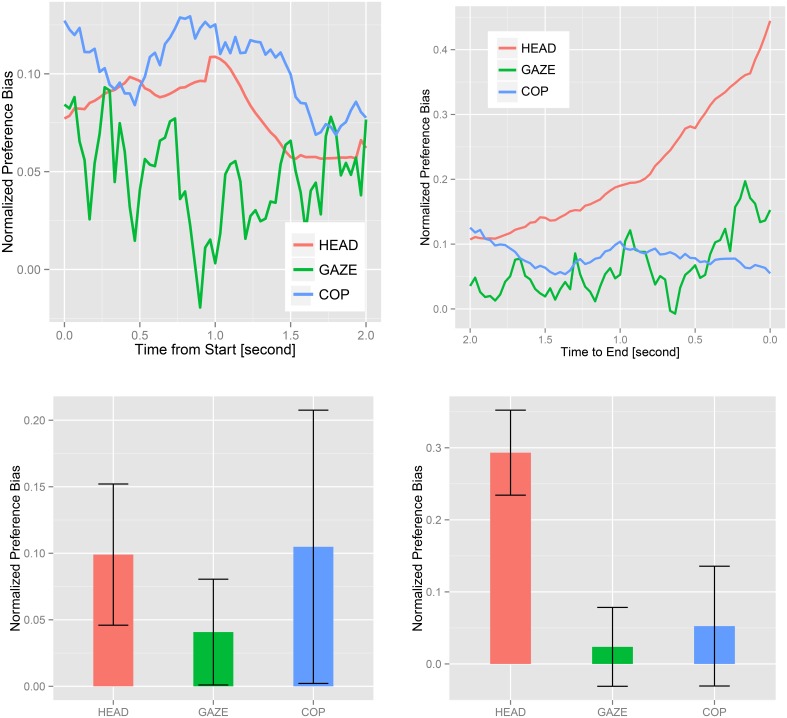
**Top:** plots of normalized *preference bias* aligned at the beginning time (left) and at the ending time (right) averaged over all samples in *case 1*. **Bottom**: bar-plots of the samples averaged over the beginning (left) and the ending (right) 1 s with standard errors in *case 1*.

Figure 6 shows the normalized *preference biases* of head orientation, gaze, and COP averaged over all subjects in *case 1*. We tested if the signals were biased toward the item by one-sample, one-sided Student's *t*-test or Wilcoxson's signed rank test after normality test. The data were averaged over corresponding trials per subject. In the 1 s from trial beginning, there was significant bias in the head orientation (*p* = 0.008) in *case 1* but not in *case 2* (*p* = 0.1). The mean of the normalized COP seemed greater than that of head orientation (shown in the top-left figure of Figure 6), but the test result was not significant (*p* = 0.4). Also in the ending 1 s, statistical significance appeared only in head orientation (*p* = 0.001 for *case 1* and *p* = 0.002 for *case 2*). There were no significant effects (*p* > 0.05) in gaze and COP for two timing conditions in both *cases*. The degrees of freedom of the tests above were again 14 in *case 1* and 15 in *case 2*.

To compare the differences among the three signals, we employed the Steel-Dwass method for head orientation, gaze, and COP. In the beginning 1 s, there were no significant differences in any pairs in both *cases* (*p* > 0.05). In the ending 1 s, a statistical significance was found between head orientation and gaze (*p* = 0.01 for *case1* and *p* = 0.02 for *case 2*). Head orientation and COP were also different (*p* = 0.01 for *case 2* and *p* = 0.001 for *case 2*). We could not find differences in any other pairs.

## 4. Discussion

The main findings of our study were (1) when the body orientation is straight, the head orientation is biased toward the selected item in the early stage of choice, i.e., beginning 1 s, and (2) the head orientation is biased more toward the selected item than gaze and COP in the late stage, i.e., ending 1 s.

The results of the early stage showed that the head orientation had a *preference bias* in *case 1* in which subjects stood straight toward the shelf, but not in *case 2*. This implies that observing early behavior of the head orientation allows to predict human preference at least when subjects stand straight toward the shelf. Although multiple comparison did not show difference in any pairs, head orientation could be a better indicator of human preference in an early stage of human choice.

On the other hand, the head orientation had a strong *preference bias* in the late stage while other signals were not biased significantly. The result of multiple comparison showed that the head orientation was more biased toward the preferred items than the gaze and the COP. The gaze behavior in the late stage did not show gaze *cascade effect* contrary to the previous studies (Shimojo et al., 2003; Atalay et al., 2012). The difference in head-immobilization could be an explanation of this difference. However, because gaze *cascade* was observed both in a head-immobilized condition (Glaholt and Reingold, 2009) and in a free-moving condition (Bee et al., 2006), we speculate that head immobilization does not affect the behaviors in gaze at least in a qualitative way. Therefore, gaze *cascade* should have occurred also in our experiments, perhaps after the decision timing we defined. Another possibility is the difference in timing between gaze and head. While previous studies asked the subjects to press a button (*timing A*) when they decided their minds, in our study, we defined the end of trial as when the subjects started moving their arms (*timing B*). *Timing A* comes a little later than *timing B* if *timing A* corresponds to the timing when the subjects finished pointing. In fact, we observed marginally significant (*p* = 0.06, Student's *t*-test for *case 1*) and significant (*p* = 0.009) preference bias in gaze for the last half second and for the half second after the decision timing, respectively. Therefore, in the late stage of choice, head would orient to the selected item earlier than gaze in the current free-moving setting, although there is still a possibility that finger-pointing movements affected the head and gaze behaviors.

From the marketing point of view, the results on head orientation suggested that observing a customer's head behaviors would be helpful in both understanding their preferences and promoting sales. One application could be to use smart digital signage on the shelves in supermarkets or kiosk stores. The signage would work as follows: First, it observes a customer's head movement using a camera with a face-tracking method and then it performs a sales promotion based on the preference estimation before the customer reaches his or her hand to an item. The estimation is expected to be more accurate if the customers stand straight toward the shelf, such as in front of a showcase or a cashier. Those systems would be also useful in terms of protecting personal information because the prediction method proposed in this paper does not use any previous information, such as purchase history, age and sex, about the customer except for the head-movement information which can be obtained and abandoned immediately.

There are several limitations in our study. First, the number of subjects was limited (*n* = 15 or 16). Second, the present analysis did not consider personal physical tendencies; for example, one's consistent or preferred head orientation might be toward left or right. This is not *body-orientation bias* and may decrease the accuracy of prediction. This problem is about personal tendencies which are difficult to identify on the fly, but could be learned after several trials. Third, no money was involved in our experiment. The result showed that there was no significant difference in the response times for snacks and clothings. This might be because subjects were only asked to choose from two alternatives without considering cost or value trade-offs. In the future, we hope to conduct a new real-shop experiment in which values of the alternatives are different and real money is involved. Finally, the other signals pertaining to body, e.g., torso orientation or waist orientation, were not measured in this study. Although there was no information in COP, the orientation of torso may have information about preference similar to that in head orientation. We also plan to investigate the relationship between preference and those signals.

## 5. Conclusion

We conducted a forced-choice task of two non-durables employing 16 healthy adults and found that there was a significant bias in the head orientation toward the item which was later chosen. A *preference bias* was observed in the head orientation in the beginning 1 s when the subjects stood straight toward the shelf, while we did not see those biased in the gaze and the COP. In the ending 1 s, only the head orientation had a *preference bias* and was more biased than other signals. Manipulating body angle did not influence the choice. Conducting experiments in real shops involving real money and investigating the relationships between other behaviors and choice are our future work.

## Author contributions

The first author, HF, contributed the concept, experimental design, data acquisition, analysis and interpretation, and report drafting. TS supervised the whole process and provided critical revisions to the manuscript.

### Conflict of interest statement

The authors declare that the research was conducted in the absence of any commercial or financial relationships that could be construed as a potential conflict of interest.

## References

[B1] Atalay A. S. Bodur H. O. Rasolofoarison D. (2012). Shining in the center: central gaze cascade effect on product choice. J. Consum. Res. 39, 848–866. 10.1086/665984

[B2] Bartlett H. L. Ting L. H. Bingham J. T. (2014). Accuracy of force and center of pressure measures of the wii balance board. Gait Posture 39, 224–228. 10.1016/j.gaitpost.2013.07.010 23910725 PMC3842432

[B3] Bee N. Prendinger H. André E. Ishizuka M. (2006). Automatic preference detection by analyzing the gaze ‘cascade effect’, in 2nd Annual Conference on Communication by Gaze Interaction (COGAIN 2006) (Turin, Italy).

[B4] Bird G. D. Lauwereyns J. Crawford M. T. (2012). The role of eye movements in decision making and the prospect of exposure effects. Vis. Res. 60, 16–21. 10.1016/j.visres.2012.02.014 22425778

[B5] Carpenter R. Williams M. (1995). Neural computation of log likelihood in control of saccadic eye movements. Nature 377, 59–62. 7659161 10.1038/377059a0

[B6] Clark R. A. Bryant A. L. Pua Y. McCrory P. Bennell K. Hunt M. (2010). Validity and reliability of the nintendo wii balance board for assessment of standing balance. Gait Posture 31, 307–310. 10.1016/j.gaitpost.2009.11.012 20005112

[B7] Funatsu N. Takahashi T. Deguchi D. Ide I. Murase H. (2013). A study on gaze estimation using head and body pose information, in Proceedings of International Workshop on Advanced Image Technology (IWAIT) (Nagoya), 231–235.

[B8] Glaholt M. G. Reingold E. M. (2009). The time course of gaze bias in visual decision tasks. Vis. Cogn. 17, 1228–1243. 10.1080/13506280802362962

[B9] Krajbich I. Armel C. Rangel A. (2010). Visual fixations and the computation and comparison of value in simple choice. Nat. Neurosci. 13, 1292–1298. 10.1038/nn.2635 20835253

[B10] Lucas B. D. Kanade T. (1981). An iterative image registration technique with an application to stereo vision, in International Joint Conference on Artificial Intelligence (IJCAI-81) (Vancouver, BC), 674–679.

[B11] Obdrzalek S. Kurillo G. Ofli F. Bajcsy R. Seto E. Jimison H.. (2012). Accuracy and robustness of Kinect pose estimation in the context of coaching of elderly population, in 2012 Annual International Conference of the IEEE, Engineering in Medicine and Biology Society (EMBC) (San Diego, CA: IEEE), 1188–1193. 10.1109/EMBC.2012.6346149 23366110

[B12] Orquin J. L. Mueller L. S. (2013). Attention and choice: a review on eye movements in decision making. Acta Psychol. 144, 190–206. 10.1016/j.actpsy.2013.06.003 23845447

[B13] Payne J. W. (1976). Task complexity and contingent processing in decision making: an information search and protocol analysis. Organ. Behav. Hum. Perform. 16, 366–387.

[B14] Rao K. S. Chandran K. R. (2013). Mining of customer walking path sequence from RFID supermarket data. Elect. Gov. Int. J. 10, 34–55. 10.1504/EG.2013.051278

[B15] Ratcliff R. Smith P. L. (2004). A comparison of sequential sampling models for two-choice reaction time. Psychol. Rev. 111:333. 10.1037/0033-295X.111.2.333 15065913 PMC1440925

[B16] Russo J. E. Leclerc F. (1994). An eye-fixation analysis of choice processes for consumer nondurables. J. Cons. Res. 21, 274–290.

[B17] Shimojo S. Simion C. Shimojo E. Scheier C. (2003). Gaze bias both reflects and influences preference. Nat. Neurosci. 6, 1317–1322. 10.1038/nn1150 14608360

[B18] Sorensen H. (2009). Inside the Mind of the Shopper: the Science of Retailing. Upper Saddle River, NJ: Pearson Prentice Hall.

[B19] Tomasi C. Shi J. (1994). Good features to track, in Proceedings of Computer Vision and Pattern Recognition, 1994 (CVPR94), 600, 593–593.

[B20] Yada K. (2011). String analysis technique for shopping path in a supermarket. J. Intell. Inform. Syst. 36, 385–402. 10.1007/s10844-009-0113-8

